# Hmgcr in the *Corpus Allatum* Controls Sexual Dimorphism of Locomotor Activity and Body Size via the Insulin Pathway in *Drosophila*

**DOI:** 10.1371/journal.pone.0000187

**Published:** 2007-01-31

**Authors:** Yesser Hadj Belgacem, Jean-René Martin

**Affiliations:** Laboratoire de Neurobiologie Cellulaire et Moléculaire (NBCM), Centre National de la Recherche Scientifique (CNRS), Unité UPR-9040, Gif-sur-Yvette, France; Ecole Normale Superieure, France

## Abstract

The insulin signaling pathway has been implicated in several physiological and developmental processes. In mammals, it controls expression of 3-Hydroxy-3-Methylglutaryl CoA Reductase (HMGCR), a key enzyme in cholesterol biosynthesis. In insects, which can not synthesize cholesterol *de novo*, the HMGCR is implicated in the biosynthesis of juvenile hormone (JH). However, the link between the insulin pathway and JH has not been established. In *Drosophila*, mutations in the insulin receptor (InR) decrease the rate of JH synthesis. It is also known that both the insulin pathway and JH play a role in the control of sexual dimorphism in locomotor activity. In studies here, to demonstrate that the insulin pathway and HMGCR are functionally linked in *Drosophila*, we first show that *hmgcr* mutation also disrupts the sexual dimorphism. Similarly to the InR, HMGCR is expressed in the *corpus allatum* (*ca*), which is the gland where JH biosynthesis occurs. Two p[hmgcr-GAL4] lines were therefore generated where RNAi was targeted specifically against the HMGCR or the InR in the *ca*. We found that RNAi-HMGCR blocked HMGCR expression, while the RNAi-InR blocked both InR and HMGCR expression. Each RNAi caused disruption of sexual dimorphism and produced dwarf flies at specific rearing temperatures. These results provide evidence: (i) that HMGCR expression is controlled by the InR and (ii) that InR and HMGCR specifically in the *ca*, are involved in the control of body size and sexual dimorphism of locomotor activity.

## Introduction

The high degree of similarity displayed among species by the insulin signaling pathway highlights its importance in developmental and physiological processes. In mammals, insulin receptor (InR) and insulin like growth factor receptor (IGFR) share the same signaling pathway components and insulin receptor substrates (IRS), which mediate functions, such as growth [1], [2], fertility and glucose homeostasis [3]. They also modulate several metabolic pathways, such as cholesterol biosynthesis [3], [4] or lipid metabolism [5]. In lower organisms like *Drosophila*, seven genes coding for insulin like ligands have been described [6]. Four of them are synthesized in neurons of the *Pars Intercerebralis* (*PI*) called Insulin Producing Cells (IPCs) [6]–[9]. In contrast, only one insulin like receptor (InR) has been described [10] with a conserved structure. Functionally, in comparison to mammals, its action is similar, during development, to insulin like growth factor receptor (IGFR) [8], [11]–[18], while in adult stage, to the endocrine function of the InR [19]–[21]. Indeed, mutations affecting InR or *chico*, the IRS homologue, or ablation of insulin producing cells (IPCs), cause growth retardation [12], [15], [16], reduce body and organ size [6], [8], [16], [22], increase sugar level [8], [13]–[15], [21], and longevity [15], [16], [23]. These phenotypes resemble those obtained for IRS-1 [24] or IRS-2 knockout mice [25].

The highly conserved insulin pathway between *Drosophila* and mammals, and the wide variety of genetic and molecular tools of *Drosophila*, gives us the opportunity to use this relatively simple model to identify new roles of the insulin pathway. Indeed, recent studies demonstrated that the insulin pathway is an integrative system adapting growth to food availability [7], [26], [27]. Additionally, Wu and collaborators implicated InR in food intake and noxious food aversion [19], [20]. In a previous study, we also reported a new role for insulin pathway in *Drosophila*, by showing its implication in the control of a sexual dimorphism in locomotor activity [21]. This sexually dimorphic behavior comprises differences in the temporal pattern of locomotor activity, represented by a different number of activity/inactivity periods (or start/stop number) between males and females when flies are freely walking during a determined period (5 hours) [21], [28]–[30].

A disruption in the insulin pathway either in IPCs or at the InR level abolishes sexual dimorphism [21]. In an earlier study, we found that the Juvenile Hormone (JH), one of the main hormones in insects, is involved in the control of start/stop number [28]. JH is synthesized in the *ca* [31], while the 3-Hydroxy-3-Methylglutaryl Co Enzyme A Reductase (HMGCR) [31] is the key step in JH biosynthesis. Additionally, ectopic application of Fluvastatin, an HMGCR inhibitor, abolishes sexual dimorphism [28]. Interestingly, we previously found that InR is expressed in the *ca* [21], while Tatar and collaborators demonstrated that a mutation affecting the InR leads to a decrease in the JH level [15]. Together, these data suggest that there is a functional link between the insulin pathway and JH synthesis and/or release in the *ca*. We therefore hypothesized that HMGCR could be the link between the insulin pathway and JH in the *ca*.

In mammals, HMGCR is a transmembrane glycoprotein anchored in the smooth endoplasmic reticulum [32] and is principally found in liver tissues [33]. It catalyzes the production of mevalonate, which represents the rate limiting step of cholesterol biosynthesis in the liver [34]. Tight control on cholesterol production is critical in physiology, since defects reducing cholesterol synthesis leads to the Smith-Lemli-Opizt syndrome [35]. Alternatively, over production causes a predisposition to atherosclerotic vascular diseases [36]. HMGCR has also been implicated in other processes like embryogenesis [37] and cancer [38], [39]. The reductase is continuously transcribed and is regulated by a number of factors, including negative feed back from cholesterol [40], or remarkably by insulin that strongly stimulates HMGCR synthesis [40]–[42]. Regulation involves a family of helix-loop-helix transcription factors, called sterol response element binding protein (SREBP) and particularly SREBP-1c [42], [43].

In insects, cholesterol is not synthesized *de novo* [44]. However, in the *ca*, HMGCR catalyzes the synthesis of mevalonate, the precursor of the JH family components [31]. In *Drosophila, hmgcr* gene (also named *columbus* (*clb*)), has been cloned and is implicated in germ cell guidance during development [45], as well as further in zebrafish [46]. HMGCR also plays a role in the potentiation of hedgehog signaling [47] as well as in neurodegenerative disease [48]. However, little is known about its regulation. Although a homologue of SREBP has been identified in *Drosophila* (HLH106) and is expressed in the *ca* [49], [50], there is still no clearly established link between HMGCR and this transcription factor.

Here, we show that a mutation affecting the *hmgcr* gene in *Drosophila*, abolishes sexual dimorphism in locomotor activity. We also show that this phenotype can be rescued by over expression of *hmgcr* specifically in the *ca*. For the latter studies, we generated two independent p[hmgcr-GAL4] lines to specifically drive either a RNAi-HMGCR or an RNAi-InR in the *ca*. In both cases, we show that locomotor behavior mimics the *hmgcr* mutant (lack of the sexual dimorphism), highlighting the role of the *hmgcr* and the insulin pathway in the *ca*. Furthermore, we also show that silencing *inr* gene within the *ca* results in a reduction of HMGCR, suggesting a transcriptional control exerted by insulin signaling pathway on the HMGCR in the *ca*. Finally, targeting either RNAi-InR or RNAi-HMGCR specifically in the *ca*, yields dwarf flies, suggesting a very specific action of InR and HMGCR within the *ca*, in the control of development.

## Results

### Sexual dimorphism in locomotor activity is abolished in the *hmgcr* mutant

**Figure 1:**
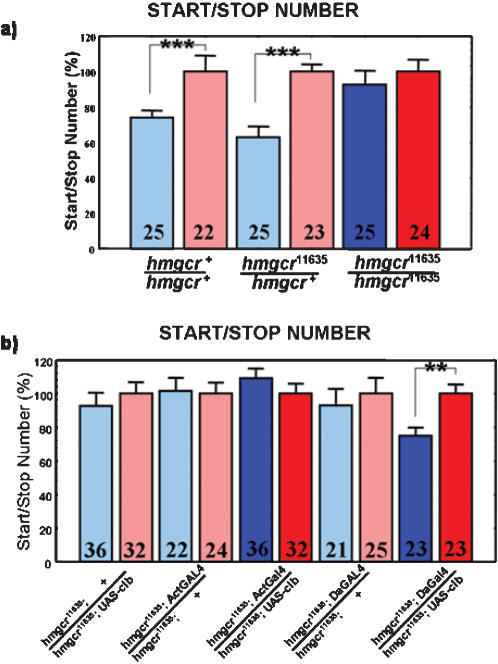
The sexual dimorphism in locomotor activity is disrupted in *hmgcr* mutant. a) Flies homozygous for a P element inserted in the 3′ region of the *hmgcr* gene (*hmgcr^11635^/hmgcr^11635^*) do not exhibit a sexually dimorphic start/stop number. Flies heterozygous for this P element insertion (*hmgcr^11635^*/CS) behave like wild-type controls Canton-S flies. For all graphics representing the start/stop number in this study, blue and red are males and females respectively, while the number in the boxes indicates the number of flies recorded for each genotype. CS = Canton-S. Mean±SEM is represented and all statistical tests are done using an Anova-Manova test (Statistica software). * p<0.05; ** p<0.01; *** p<0.001. b) Targeted expression of p[UAS-*clb*] under the control the p[*da*-GAL4] driver in flies homozygous for the *hmgcr^11635^* allele (p[*da*-GAL4]; *hmgcr^11635^*/UAS-*clb, hmgcr^11635^*) is sufficient to rescue both the lethality and the sexual dimorphism, observed in *hmgcr^11635^/hmgcr^11635^* flies. However, the p[*act*-GAL4] driver (p[*act-*GAL4]; *hmgcr^11635^*/UAS-*clb, hmgcr^11635^*) rescue the lethality, but not the sexual dimorphism.

The number of activity/inactivity phases, equivalent to the start/stop number, has been shown to be different between males and females in normal wild-type (Canton Special (CS)) flies [30]. Application of Fluvastatin, an HMGCR inhibitor [51] abolishes the sexual dimorphism [28]. In an attempt to determine whether HMGCR is directly involved in the control of this dimorphism, we quantified the locomotor activity of flies carrying a homozygous mutation affecting the gene *hmgcr*. Since all known *hmgcr* mutations were homozygous lethal [45], and heterozygous combinations [(P{PZ}*hmgcr^01152^*/CS) and (*hmgcr^C14.5^*/CS)] exhibit a wild-type like phenotype (data not shown), we used an hypomorphic mutation of the *hmgcr* gene, a line carrying a P element in the 3′ region of this gene (P{PZ}l(3)04684^04684^), also known as 11635 line (Bloomington Stock Center number). When reared at 24°C, the 11635 homozygous flies are also lethal. However, when reared at 19°C, 5 and 10% of males and females, respectively, which are developmentally delayed, attempt to survive until adulthood. Quantification of the locomotor activity of these flies indicates that the start/stop number for both sexes is the same (Figure 1a). However, at this stage of the study, we could not exclude that the P element affects other genes in the 3′ region of *hmgcr*. To confirm that the effects observed in the 11635 homozygous mutants are due to a perturbation of the *hmgcr* gene, we genetically rescued the phenotype by directing the expression of the the *hmgcr* gene in the 11635 homozygous mutant. In these studies, we used the yeast derived UAS/GAL4 system to specifically target the *hmgcr* cDNA: p[UAS-*hmgcr*] (also known and referred here as: UAS-*clb*) [45] with different p[GAL4] driver lines in an homozygous 11635 genetic background mutant. The pan-neural p[elav-GAL4] (elav-GAL4) [52] or the head specific fat body p[*takeout*-GAL4] (to-GAL4) [53] drivers were not able to rescue lethality. However, the p[actin-GAL4] (act-GAL4) [54] a driver known to be expressed in many tissues and cells (but not considered an ubiquitous driver), completely rescues lethality but, surprisingly, does not rescue the sexual dimorphism (Figure 1b). Finally, the p[*daughterless*-GAL4] (da-GAL4), an ubiquitous driver [55] was sufficient to rescue both lethality [partly in males (75%) and completely in females (Figure 1b)] and the start/stop number. The rescue of the locomotor activity defect in the P element 11635 mutant flies (*hmgcr^11635^* allele) confirms that this phenotype resulted from mutation to the *hmgcr* gene by the P element insertion. However, to elucidate the difference in the rescue (lethality and sexual dimorphism) obtained with those two drivers, act-GAL4 versus da-GAL4, we also looked precisely at their respective expression patterns, using the reporter transgene UAS-*gfp* (green fluorescent protein). These studies showed that GFP was found to be expressed in the *ca* under the sole control of the da-GAL4 line, but not in the act-GAL4 (data not shown), suggesting a specific role for HMGCR in the *ca* to control sexual dimorphism. These results suggest firstly, that the HMGCR is involved in the control of sexual dimorphism and secondly, that its action may be required in the *ca*.

### *hmgcr* is expressed in the fat body and the *corpus allatum* in adult flies

**Figure 2:**
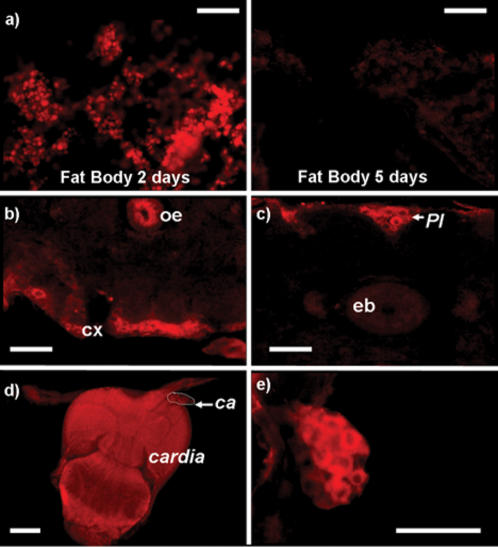
***hmgcr*** is expressed in the brain, the larval fat body, the *cardia* and the *corpus allatum*. Immunohistological staining showing the HMGCR expression in various tissues, in adult fly. a, b, c) The HMGCR is expressed in the fat body in flies younger than 4 days (a, left). In flies older than 5 days, no HMGCR expression is detectable (a, right). The HMGCR is also expressed in some neurons, principally in the cortex of the brain (cx) (b) and some cells of the *pars intercerebralis* (*PI*) (c). oe: eosophagus, eb: ellipsoid-body. d, e) In the body, the reductase is detected in the *cardia* (d) and in the *corpus allatum* (*ca*) (e). For a negative control of the HMGCR staining, see Figure 5b, in comparison with figures 5a, c, d, e and g. Scale bar = 25 µm.

To determine the expression pattern of *hmgcr*, we performed immunostaining. In mammals, the enzyme has been found in a wide variety of tissues including brain, testis and liver [40]. Polyclonal antibodies raised against the human HMGCR [33] were used to determine the expression pattern of HMGCR in *Drosophila* tissues. First, we showed that the reductase is expressed in residual larval fat body (Figure 2a), which corresponds to residual adipocytes inheritated from larval stages that disappear in flies within approximately three days after hatching. HMGCR is also detected in some cells at the periphery of the brain (Figure 2b and 2c). The presence of this enzyme in the brain has already been indirectly suggested by Tschäpe and collaborators [48]. These authors have shown that HMGCR could play a role in neuronal tissues. Indeed, the ectopic expression of the reductase, under the control of the pan-neural driver, elav-GAL4, in flies carrying a mutation leading to neurodegenerescence, is sufficient to rescue the lethal phenotype. In Figure 2c, we can see an immunoreactive cluster of cells located in the *pars intercerebralis* (*PI*). Outside the head, the reductase is also found to be expressed in the digestive tract, especially in the *cardia* (Figure 2d). Finally, the HMGCR is also expressed in the *ca* (Figure 2e), in accordance with previous results suggesting a role for the reductase in this tiny gland.

### Identification of *hmgcr* regulatory sequences and genesis of two p[hmgcr-GAL4] lines

**Figure 3:**
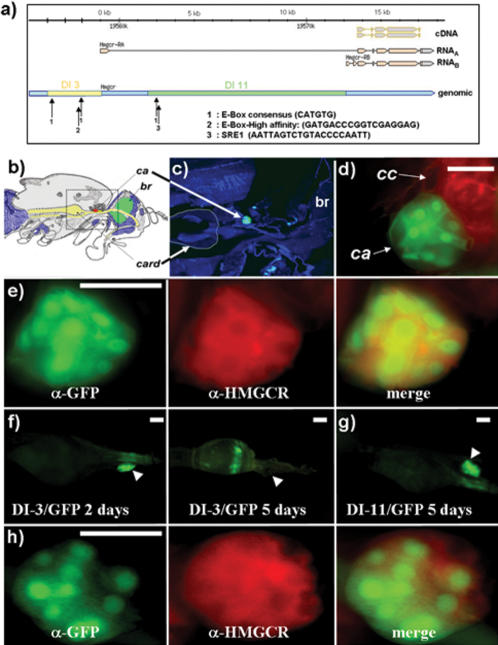
Genesis and expression pattern of the two transgenic p[hmgcr-GAL4] lines. a) Promoters and genomic regions of *hmgcr* gene. Two mRNAs are transcribed (mRNA_A_ and mRNA_B_) under the control of two distinct promoter regions. After splicing, the encoding regions of the two cDNA are identical. A computer analysis of the genomic DNA region has allowed to find two E-Box consensus sequences (CATGTG) [63] localized at −3194 to −3188 and −1315 to −1309, and one E-Box High affinity (GATGACCCGGTCGAGGAG) [64] localized at −2051 to −2033, comprised in a region of 3,8 kb just upstream of the first exon of *hmgcr*. We then hypothesized that this region could control the RNA_A_ transcription, and cloned and used it to generate the p[DI-3-GAL4] (DI-3) line. To generate the p[DI-11-GAL4] (DI-11) line, we cloned a fragment of 10,6 kb of the first intron (+2733 to +13525) in which we found an E-box consensus [63] (+2911 to +2916) and one SRE1 (AATTAGTCTGTACCCCAATT) [64] (+3551 to +3570). We hypothesized that this region could control the RNA_B_ transcription. b) Schematic lateral view of the head and thorax of *Drosophila*. card: *cardia*; *ca*: *corpus allatum*; br: brain. c–h) The two p[DI-GAL4] lines (DI-3 and DI-11) drive the expression of GFP in the *ca*. c) At low magnification (10X), the *ca*, in green, is detected by using primary antibodies against GFP and secondary antibodies labeled with FITC (green) in flies DI-3/GFP. The background is artificially colored in blue. d) The *corpus cardiacum* (*cc*) is a structure located near the *ca*. To confirm that the GFP is expressed in the *ca*, the *corpus cardiacum* (*cc*) was stained using an anti-AKH antibody [56] directly conjugated to the rhodamine (red). Thus, we can visualize the *ca* in green and the *cc* in red. e) The GFP (left, green) and HMGCR (middle, red) are detected with primary and secondary antibody. Those last are labeled respectively with FITC (green) or Cy3 (red). When merged, we can see that the GFP and HMGCR are colocalized (right panel: yellow), confirming that the DI-3 drives the GFP in the same cells of the *ca* that express the HMGCR. f) Dissection of the *cardia* and the *ca* from DI-3/UAS-*gfp* flies of different ages and observed directly under a binocular lamp fit with a green fluorescent filter (Leica, MZ FLIII). The GFP is driven by DI-3 in the *ca* in a temporal dynamic pattern. In two days old adult flies (left panel), the GFP is well detectable in the *ca* (arrowhead), but not anymore in 5 days old flies (right panel). However, in 5 days old flies, the GFP is detectable in a thin layer of the *cardia*. g) Dissection of *cardia* and *ca* from (DI-11/UAS-*gfp*) directly observed under a binocular lamp fit with a green fluorescent filter (Leica, MZ FLIII). DI-11 also drives the expression of GFP in the *ca* (arrowhead), and this expression is permanent in adult flies. h) GFP (left) and HMGCR (middle) are detected respectively with primary antibodies against GFP and HMGCR and revealed with secondary antibodies respectively labeled with FITC (green) and Cy3 (red). When merged, we can see that the GFP and HMGCR are colocalized (right panel: yellow), confirming that the DI-11 drives the GFP in the same cells of the *ca* that express the HMGCR. Scale bar = 25 µm.

To date, no specific GAL4-driver line has been reported to be specifically expressed in the *ca* in adult fly. Since we found that *hmgcr* is expressed in the *ca*, suggesting a specific role in this structure, we generated two p[hmgcr-GAL4] lines, in which the putative genomic DNA promoter sequence specifically drives the GAL4 gene. The *Drosophila hmgcr* gene is composed of 8 exons, in which 5 are coding exons. Two mRNA are transcribed according to two different initiation sites (Figure 3a). RNA_A_ (3,972 kb) comprises the first, but not the second and third exons. The RNA_B_ (3,833 kb) starts only with the second exon. Both mRNA share the other three encoding exons. In order to construct two p[GAL4] lines corresponding respectively to the two putative mRNA promoters, we first selected a sequence of 3,8 kb upstream to the first exon initiation site, and secondly, a sequence of 10,8 kb upstream to the second exon. Each sequence was cloned and fused upstream to the coding sequence of the yeast GAL4 transcription factor gene. In these two sequences, based on the already identified mammalian sequence, specific cis-acting transcription factor recognition sites were identified (Figure 3a), especially E-boxes and SRE (Sterol Responding Elements) that are known to be the targets of SREBP (Sterol Responding Element Binding Protein). The two cloned constructs were then used to generate two independent and specific p[hmgcr-GAL4] transgenic lines called DI-3 and DI-11, corresponding respectively to the RNA_A_ and RNA_B_ promoters. In an initial step, we determined if the two lines were functional. Using the UAS-*gfp* as reporter, we determined their expression pattern. Two days old DI-3/UAS-*gfp* flies were found to express the reporter gene *gfp* in the residual larval fat body (data not shown). GFP was also detected in some *PI* cells (data not shown) but not in other brain neurons. Moreover, a strong expression of the reporter gene was found in the *ca* (Figure 3c). The *ca* is a very small gland located near the *corpus cardiacum* (*cc*) and the *cardia*, a compartment of the digestive tract (Figure 3b). To confirm that the observed signal corresponds to the *ca*, a double immunostaining against GFP and the Adipokinetic Hormone (AKH) [56] was performed (Figure 3d). AKH is known to label specifically the *cc* [56], [57], allowing the presence of GFP in the *ca*. In a second step, double immunostaining against GFP and HMGCR was performed (Figure 3e), allowing colocalization of the two proteins in *ca* cells, to be shown. Thus, this expression pattern confirms that the 3,8 kb length promoter cloned fragment is sufficient to drive expression in the *ca, PI* and larval residual fat body cells. However, the previously determined endogenous HMGCR expression pattern does not fully overlap with the DI-3 driver expression pattern. Notably, in DI-3/UAS-*gfp* flies older than 4 days, a signal is detectable in a thin layer of the *cardia*, but is no longer found in the *ca* (Figure 3f), whereas GFP expression is still found in *PI* cells (data not shown). It is likely that the residual fat body cells are not detected because of the lack of these cells in 5 day old flies. These results show that DI-3 presents a temporally dynamic expression pattern. This also tends to suggest distinct roles for HMGCR in some tissues of young and older adults. By contrary to DI-3, DI-11 has a very stable temporal expression pattern. The reporter gene UAS-*gfp* driven by DI-11 shows a strong expression in the* ca* (Figure 3g). As for DI-3, a double immunostaining reveals that GFP and HMGCR are colocalized in the same cells of the *ca* (Figure 3h). In brief, DI-3 and DI-11 drive reporter gene expression in a pattern strictly included within the previously shown endogenous HMGCR expression pattern, as revealed by the immunostaining. The DI-3 follows partially and temporally the expression of the endogenous gene, while the DI-11 seems to follow permanently the expression in the *ca*. These two lines are therefore valuable tools to specifically target any reporter gene in the *ca*, so that studies on the putative role of HMGCR in this specific tiny gland can be undertaken.

### Targeted expression of *hmgcr* cDNA in the *corpus allatum* rescues the *hmgcr* mutant phenotype

**Figure 4:**
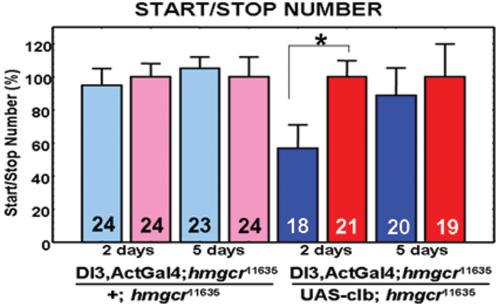
**Targeted expression of the HMGCR in the** *corpus allatum* rescues the *hmgcr* mutant phenotype. p[DI-3-GAL4] plus p[act-GAL4] in the same flies are sufficient to rescue the sexual dimorphism disrupted in hmgcr^11635^/hmgcr^11635^ flies (DI-3, act-GAL4; hmgcr^11635^/UAS-*clb*; hmgcr^11635^). This suggests a specific role for the HMGCR in the *ca* to control the sexual dimorphism. Interestingly, this rescue occurs in two days old flies, but not in the same flies of 5 days old.

We made the hypothesis that the lack of the sexual dimorphism in locomotor activity in the *hmgcr^11635^* mutant could be due specifically to the lack of the *hmgcr* in the *ca*. First, driving UAS-*clb* with DI-3 in the homozygous mutant background (DI-3; UAS-*clb, hmgcr^11635^*/*hmgcr^11635^*) was not sufficient to rescue lethality at 24°C. As we previously observed that act-GAL4 driving UAS-*clb* was sufficient to allow (*hmgcr^11635^*/*hmgcr^11635^*) flies to survive until adulthood, but not to rescue the sexual dimorphism in locomotor activity, we combined these two driver lines (act-GAL4 and DI-3) in the same fly, to drive *hmgcr* gene expression. Interestingly, the presence of the two drivers, act-GAL4 and DI-3, in the mutant genetic background (*hmgcr^11635^*/*hmgcr^11635^*) was sufficient to rescue the sexual dimorphism. Indeed, figure 4 shows that 2 days old males and females bearing both act-GAL4 and DI-3 have a significantly different start/stop number, compared to control flies, confirming that DI-3 is sufficient to rescue the sexual dimorphism. Most interesting was the observation that the expression of DI-3 disappeared after approximately 4 days. After the first locomotor activity quantification, flies were re-collected and maintained on normal food medium during 3 additional days, after which locomotor activity was again recorded and the start/stop numbers determined. Figure 4 shows that these five days old flies exhibited a mutant phenotype: males and females start/stop number was similar. This result concurs with the observed expression pattern of the *gfp* transgene under the control of DI-3, suggesting that before 3 days, *hmgcr* transgene is expressed in both the *ca* and the rest of the body (comprising the *PI* neurons), while after 4 days old, the transgene, driven only by act-GAL4 promoter, is not expressed anymore in the *ca*. This result suggests that HMGCR plays a role in the sexual dimorphism, by acting specifically within the *ca*. However, due to the reporter gene expression pattern of DI-3 in some other neurons and tissues, we can not exclude if *PI* and/or larval fat body cells could also be implicated in the control of start/stop number.

### Directed expression of RNAi-HMGCR in the *corpus allatum* abolishes sexual dimorphism and mimics the *hmgcr* mutant phenotype

**Figure 5:**
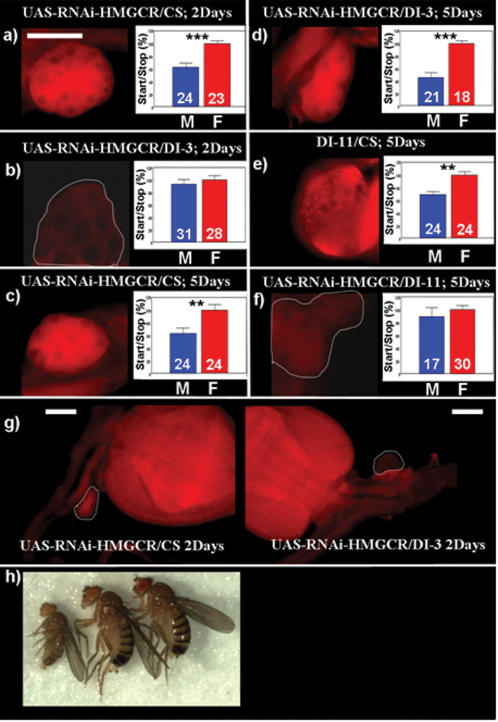
Directed expression of p[UAS-RNAi-HMGCR] specifically in the *corpus allatum* mimics the *hmgcr* mutation, by disrupting the sexual dimorphism. For all panels (a–g): immunostaining using a primary antibody raised against the human form of the HMGCR, revealed with a secondary antibody labeled with Cy3 (red). a–d) Targeting UAS-RNAi-HMGCR specifically in the *ca* yields to the lack of the HMGCR protein, and leads to the disruption of the sexual dimorphism. This effect is completely reversible. In 2 days old DI-3/UAS-RNAi-HMGCR flies (b), HMGCR protein is not detectable and the number of start/stop is identical between males and females compared to controls (a) (2 days old UAS-RNAi-HMGCR/CS flies). Interestingly, in 5 days old flies, both DI-3/UAS-RNAi-HMGCR (d) and UAS-RNAi-HMGCR/CS (c) the HMGCR is detectable and the start/stop number is sexually dimorphic. This is in agreement with the temporal expression pattern driven by the DI-3. Additionally, this result strongly suggests the reversibility of the RNAi effect. N.B: results from b) and d) come from the same flies, recorded at 2 and 5 days old respectively. e, f) Expressing the UAS-RNAi-HMGCR in the *ca* using the DI-11 line (DI-11/UAS-RNAi-HMGCR) also leads to the lack of the HMGCR product (f) and disrupts the sexual dimorphism compared to controls flies (e). g) The HMGCR is expressed both in the c*ardia* and the *ca* (left panel) in control flies (2 days old UAS-RNAi-HMGCR/CS flies), whereas in 2 days old (DI-3/UAS-RNAi-HMGCR) flies, the HMGCR is detected only in the *cardia* (right panel). This last staining serves as a positive control, to demonstrate that the DI-3 drives the UAS-RNAi-HMGCR only in the *ca*. h) Expressing the UAS-RNAi-HMGCR in the *ca* under the control of DI-11 leads to a strong lethality when flies are reared at 24°C. Moreover, the only few females that survived are dwarf (left: dwarf female DI-11/UAS-RNAi-HMGCR, middle: female control UAS-RNAi-HMGCR/CS] and right: female CS). Scale bar = 25 µm.

To determine more precisely the tissues where HMGCR is required to promote the sexual dimorphism in locomotor activity, we used an interferential RNA (RNAi) against the *hmgcr* gene. For each experiment, DI-3 line in combination with one of the two p[UAS-RNAi-HMGCR] lines (10367R1 and 10367R3, inserted on the chromosome III and II respectively; a generous gift from R. Ueda, NIG, Japan) were used to avoid non specific P element insertion side effect. We found that flies carrying the UAS-RNAi-HMGCR driven by either the da-GAL4 ubiquitous driver or by the act-GAL4 driver, yields lethality to the second instars larva. Although we can not exclude a non specific toxic effect, this phenotype is comparable to lethality induced by *hmgcr* mutations. Compared to controls: DI-3/CS and/or UAS-RNAi-HMGCR/CS, no HMGCR was detected in the *ca* in 2 days old flies when the UAS-RNAi-HMGCR was driven by DI-3 (Figures 5a and 5b). It is worth reiterating, that in 2 days old flies, DI-3 does not drive expression of UAS-*gfp* in the *cardia* (see Figure 3f) and we can presume that the expression pattern is the same for UAS-RNAi-HMGCR. Indeed, the *cardia* (Figure 5g) is sufficiently immunoreactive independently of the presence of the UAS-RNAi-HMGCR transgene. This result confirms that HMGCR is not detectable in tissues simultaneously expressing the GAL4 driver and the UAS-RNAi-HMGCR and thus, validates the specificity of action of the latter. In accordance with previous results, two days old flies without HMGCR expression in the *ca* (DI-3/UAS-RNAi-HMGCR) did not show the sexual dimorphism in locomotor activity (Figure 5b). Furthermore, UAS-RNAi-HMGCR/CS control flies expressing HMGCR in the *ca*, harbor a wild type phenotype, as the sexual dimorphism is present (Figure 5a). As for the former experiment, all the 2 days old recorded flies were kept for further re-evaluation, and then, placed in a new food medium for 3 days before their locomotor activity was once again quantified. As expected, all five days old flies (controls UAS-RNAi-HMGCR/CS or DI-3/UAS-RNAi-HMGCR) present a sexual dimorphism comparable to a wild-type phenotype (Figures 5c and 5d). Indeed, as previously observed with the UAS-*gfp*, DI-3 does not drive UAS-RNAi-HMGCR in flies older than 4 days. This result together with the data supporting the presence of HMGCR protein in the *ca* (Figure 5d), suggests that the action of the RNAi is at least partly reversible. However, we can still not exclude a possible action of HMGCR in *PI* and/or residual larval fat body cells. To rule out this possibility, we used DI-11 line to drive UAS-RNAi-HMGCR in the *ca*. When reared at 24°C, DI-11/UAS-RNAi-HMGCR larvae are smaller than control (half size) (Figure 5h) and a strong lethality is observed in third instars larvae, as very few adults survive: less than 1% and females only. Fortunately, when reared at 19°C, 10% and 1% of expected adult females and males, respectively, survive and have normal weight and size. As we can expect for these flies, a lack of sexual dimorphism is observed in correlation with the absence of HMGCR expression in the *ca* (Figure 5f) compared to the relevant controls (Figure 5e). These results are in accordance with those obtained using the DI-3 line. To exclude possible contributions from other tissues in the sexual dimorphism, and more particularly the involvement of the HMGCR expressed in *PI* and/or residual larval fat body cells, we used two other GAL4 driver lines, p[GAL4]C316 (C316) and elav-GAL4 to drive UAS-RNAi-HMGCR. C316 induces UAS-*gfp* expression in the larval fat body, in the *cardia* and in DPM neurons (data not shown) [58], while elav-GAL4 is a *pan*-neural GAL4-driver [52]. Flies from either elav-GAL4/UAS-RNAi-HMGCR or C316/UAS-RNAi-HMGCR display a wild type start/stop number compared to controls (data not shown). Altogether, these results strongly suggest that HMGCR expression in the *ca* is necessary for flies to present the sexually dimorphic locomotor activity.

### Directed expression of RNAi-InR in the *corpus allatum* abolishes the sexual dimorphism and mimics the *hmgcr* mutation

**Figure 6:**
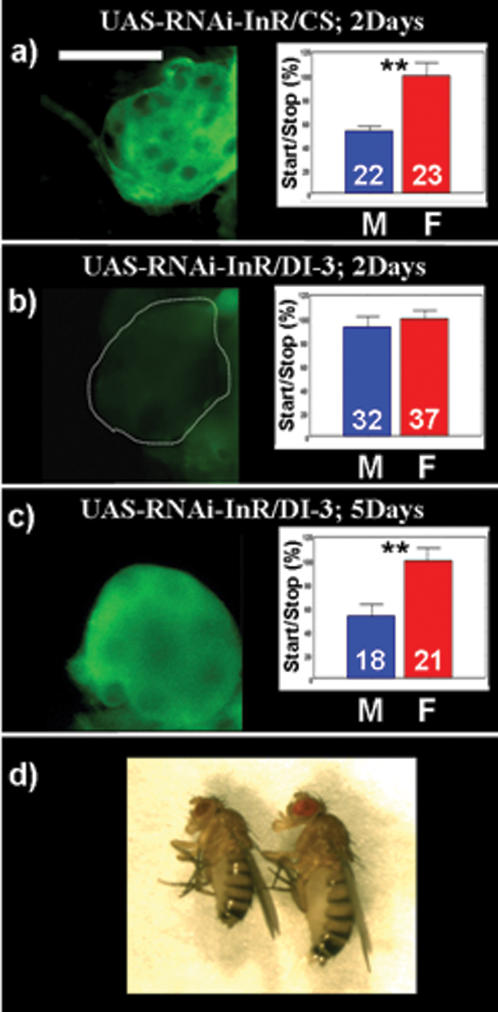
Directed expression of p[UAS-RNAi-InR] specifically in the *corpus allatum*, mimics the *hmgcr* mutation. For all panels (a–c): immunostaining using a primary antibody raised against the human form of the InR, revealed by a secondary antibody labeled with FITC (green). a–c) Targeting the UAS-RNAi-InR specifically in the *ca* blocks the InR expression and disrupts the sexual dimorphism. This effect is completely reversible. In 2 days old DI-3/UAS-RNAi-InR flies (b), InR is not detectable and the number of start/stop is identical between males and females compared to controls flies (a) (2 days old UAS-RNAi-InR/CS). Interestingly, in 5 days old DI-3/UAS-RNAi-InR flies (c) the InR is detectable and the start/stop number is sexually dimorphic. This result corroborates the temporal expression pattern driven by the DI-3. Additionally, this strongly suggests, like for the RNAi-HMGCR, the reversibility of the RNAi-InR effect. N.B.: again here, results from b) and d) come from the same flies, recorded at 2 and 5 days old, respectively. d) Expressing the UAS-RNAi-InR in the *ca* under the control of DI-11 leads to a strong lethality when flies are reared at 24°C and 19°C. However, at 19°C, few females survive, but they are dwarf (left: dwarf female DI-11/UAS-RNAi-InR, right: female control (UAS-RNAi-InR/CS). Scale bar = 25 µm.

In a previous study [21], we reported that the insulin signaling pathway is implicated in the start/stop number control, and in parallel, that the insulin receptor (InR) is expressed in the *ca*. Using the same strategy, we used the DI-3 line to drive a RNAi against the InR gene (p[UAS-RNAi-InR]) in the *ca*. As before, the DI-3 line and two UAS-RNAi-InR lines (18402R1 and 18402R2, inserted on the chromosome II and III respectively, also a generous gift from R. Ueda, NIG, Japan) have been used to avoid non specific P element insertion side effect. Again here, flies carrying a UAS-RNAi-InR driven either by act-GAL4 or by da-GAL4 lines are lethal in the second instars larva. However, using the DI-3 driver line, we obtained flies from 2 days old (DI-3/UAS-RNAi-InR), which do not have InR expression in *ca* (Figure 6b), while the start/stop number is not different between males and females (no sexual dimorphism) compared to controls (Figure 6a). Both sexual dimorphism and InR expression are completely restored in 5 day old DI-3/UAS-RNAi-InR flies, which are comparable to control flies: DI-3/CS and UAS-RNAi-InR/CS (Figure 6c) (as previously, the same flies are recorded at 2 and 5 days old). Similarly to the UAS-RNAi-HMGCR/CS flies, UAS-RNAi-InR/CS flies are normal, suggesting a very putative restricted non specific miscellaneous expression of RNAi. Another similarity to the RNAi-HMGCR, is that the RNAi-InR effects are at least partly reversible. We previously showed that using the DI-11 in combination with the UAS-RNAi-HMGCR leads to lethality at 24°C. Also here, using UAS-RNAi-InR driven by the DI-11 yields lethality at 24°C. However, when reared at 19°C, we obtained few adult females (less than 1%) that are dwarf and die after about 5 days (Figure 6d). To confirm the specific role of HMGCR within the *ca* versus the one expressed in the *PI* or in the residual larval fat body cells, we used again the two GAL4 driver lines, C316 and elav-GAL4 to drive the UAS-RNAi-InR. In both cases, neither InR expression and/or the sexual dimorphism were affected (data not shown). In conclusion, InR expression in the *ca* seems to be necessary for establishing the sexual dimorphism.

### Insulin signaling pathway controls the sexual dimorphism via *hmgcr*

**Figure 7:**
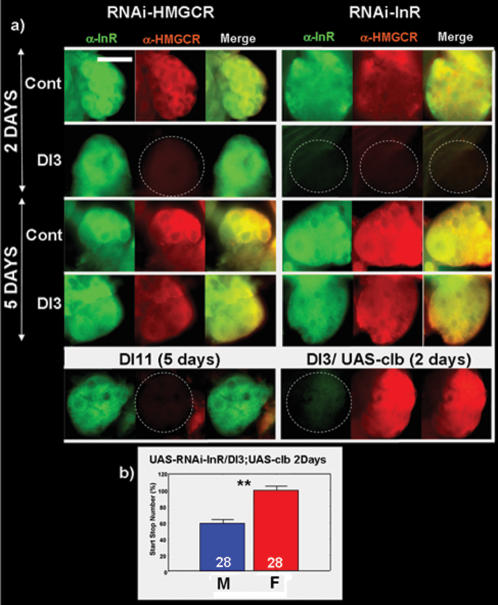
Insulin signaling pathway controls the sexual dimorphism via HMGCR. a) Immunostaining using primary antibodies against the InR and/or HMGCR, revealed by secondary antibodies labeled with FITC (green) or Cy3 (red) respectively. HMGCR and InR are colocalized in the same cells of the *ca*. Blocking the expression of the HMGCR does not influence the expression of the InR, but abolishes the sexual dimorphism. However, blocking the expression of the InR blocks the expression of the HMGCR and consequently abolishes the sexual dimorphism. This suggests that the InR controls the expression of the HMGCR within the *ca*. To test this hypothesis, we directed the expression of the UAS-*clb* concomitantly with the UAS-RNAi-InR (DI-3/UAS-RNAi-InR; UAS-*clb*). In this case, although InR is not detectable, HMGCR is present in the *ca* and the start/stop number is sexually dimorphic between males and females (b). Scale bar = 25 µm.

We have shown that InR and *hmgcr* are both expressed in the *ca*. When a RNAi-HMGCR or a RNAi-InR is driven specifically in the *ca* by DI-3 or DI-11 drivers, this leads to a disruption of the sexual dimorphism in locomotor activity. To demonstrate that the InR and HMGCR are expressed in the same *ca*'s cells, we performed a double immunostaining against HMGCR and InR. Figure 7a shows that those two proteins are colocalized. Using the DI-3 and/or the DI-11 as drivers, we first showed that expressing the RNAi-HMGCR in the *ca* does not affect InR expression (Figure 7a). Conversely, expression of an RNAi-InR completely suppresses *hmgcr* expression. Similarly to previous reports in mammalian cells [42], these results suggest a putative transcriptional control exerted by InR on *hmgcr*. Furthermore, since DI-3 drives expression of UAS-RNAi only up to 3 days, then expression of both InR and/or HMGCR, which correlates to the sexual dimorphism, is restored at 5 days. This confirms that the RNAi effects are reversible (Figure 7a). However, no direct evidence that the disruption of the sexual dimorphism could be attributed to the lack of HMGCR rather than to the InR. Indeed, both HMGCR and InR could be needed for the sexual dimorphism, but in independent pathways. In order to determine more precisely a functional link between these two proteins in relation to their respective roles in the control of sexual dimorphism, we expressed both *hmgcr* (using UAS-*clb*) and UAS-RNAi-InR under the control of the DI-3 driver. We found that InR was still undetectable whereas HMGCR was well present in the *ca* in 3 days old flies (Figure 7a) and the sexual dimorphism of these flies was determined to be wild type (Figure 7b). Taken together, these results strongly suggest, as in mammals [42], a transcriptional control of HMGCR by the insulin signaling pathway in the *ca*. In addition, InR seems to control the sexual dimorphism through the HMGCR enzyme.

## Discussion

### A specific physiological role for the HMGCR

We have shown, in *Drosophila*, by immuno-histological staining, that HMGCR is expressed in a variety of tissues, including the digestive tract, the brain and the *ca*. Moreover, this expression pattern appears to be temporally dynamic. Here, the genesis of two transgenic p[hmgcr-GAL4] driver lines (DI-3 and DI-11) corresponding to the promoter regions of the two transcripts (mRNA_A_ and mRNA_B_) respectively, have allowed different reporter genes to be targeted specifically in the *ca*, allowing more precise studies on its specific physiological role. Using the UAS-*gfp* as reporter gene, we showed that the putative promoter sequence of mRNA-a (DI-3) drives GFP expression in some cells of the *PI* within the brain. Furthermore, in young flies less than 3 days old, GFP expression is found in the digestive tract, in the residual larval fat body and in the *ca*. In contrast, DI-11 has a more restricted spatio-temporal expression pattern. Indeed, in DI-11, only oesophagus and *ca* are stained. Nevertheless, if we superimpose the expression pattern of DI-3 and DI-11, the observed pattern is included, but not fully equivalent to the endogenous HMGCR's one. Thus, this suggests that the two selected 5′ genomic DNA regions do not include the full endogenous promoter region.

To refine our study, we used two new GAL4 drivers (DI-3 and DI-11) to target an RNAi-HMGCR directly into the *ca*. No HMGCR expression was detected by immuno-histochemistry in the *ca* of 5 days old DI-11/RNAi-HMGCR flies and this effect was correlated with an equal start/stop number between males and females. Results obtained with DI-3 were more complex, but informative, as its expression pattern is temporally dynamic. Indeed, DI-3 drives RNAi-HMGCR in the *ca* in flies younger than 3 days old, and in this case, HMGCR was not detectable in *ca*. In parallel, the start/stop number was found to be the same for males and females. However, as expected, 5 days old DI-3/RNAi-HMGCR flies behave strictly as controls, since HMGCR is, *de novo*, expressed in the *ca*. Altogether, these results confirm that HMGCR expression in the *ca* is necessary for a sexually dimorphic start/stop number. In addition, RNAi effects are reversible: the same DI-3/RNAi-HMGCR flies were recorded at 2 and 5 days old. This reversibility indicates that the specific effects observed in adults after RNAi induction on locomotion are physiological rather than developmental consequences resulting from the lack of HMGCR.

### Insulin pathway controls the sexual dimorphism in the *corpus allatum*

Previous studies have shown that mutations in Insulin Receptor (InR) decrease JH level [15], [59], a hormone synthesized in the *ca* and implying the HMGCR enzyme. Moreover, we have shown, using a genetic approach, that InR is implicated in the control of the sexual dimorphism in locomotor activity [21]. Here, driving an RNAi-InR specifically in the *ca* abolishes the sexual dimorphism. Similarly to the HMGCR, these results suggest that InR is required in this tissue to promote its effect on the sexual dimorphism. Furthermore, the reversibility of the effect observed with DI-3/UAS-RNAi-InR in 5 days old flies compared to 2 days old flies, tends to exclude an IGF-like effect of InR pathway on *ca* cells during development that could alter the functional capacity of *ca*. This is the first time that a “tissue specific action” of insulin, not directly related to development or carbohydrate metabolism, is reported in *Drosophila*.

InR mutations or IPCs ablation lead to a diabetes like phenotype: trehalosemia is increased in both larvae and adults [8], [13]–[15], [21]. Interestingly, neither *hmgcr* mutant nor RNAi-HMGCR or RNAi-InR expressed in the *ca* increase haemolymph sugar level (data not shown). This reveals a new and very specific functional role for the insulin signaling pathway in the *ca*. Moreover, when driven by DI-3, RNAi-InR blocks *hmgcr* expression and this action is completely reversed in flies older than five days. Although we can not exclude an IGF like action of insulin in *ca* during development, *inr* mutations did not affect *ca* size [59] and the reversibility of RNAi effects in adult flies strongly suggest that the implication of the InR and HMGCR in such physiological processes, is dynamically active.

### Insulin pathway controls HMGCR expression in the *corpus allatum*

**Figure 8:**
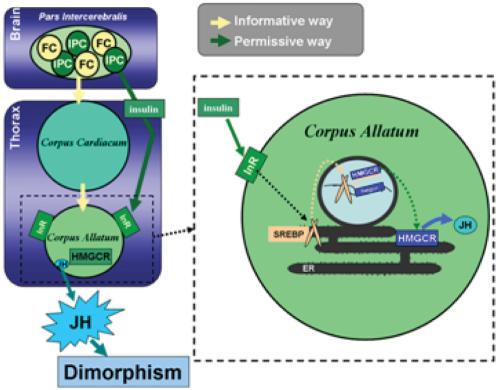
Model of the insulin pathway regulating the expression of the HMGCR in the *corpus allatum*. We hypothesize that the two different populations of cells, the Insulin Producing Cells (IPCs) and the feminising cells (FCs), located in the *pars intercerebralis* control, by two distinct ways, the JH synthesis/release by the *corpus allatum*. The informative way (in yellow), which could be feminised, might control the level or the timing of the JH release, while the permissive way (in green) might control or allow the presence of the molecular machinery, as the transcription of the HMGCR, to promote the JH synthesis. In magnification: in the *corpus allatum,* insulin binds to its receptor (InR), which activates SREBP. In turn, SREBP regulates the transcription of *hmgcr* gene.

A number of clues suggests an interaction between the insulin pathway and JH [15], [21]. In mammalian hepatocytes, HMGCR has been shown to be partially under control of insulin [40]. In *Drosophila*, the HMGCR is the key step in JH biosynthesis, which is known to occur in the *ca*. We report here that specifically silencing the *inr* gene in *ca*, under the control of DI-3 or DI-11, leads to decreased HMGCR, which abolishes sexual dimorphism. Moreover, this phenotype can be rescued by directed expression of the *hmgc*r gene within the *ca*. Similarly to results found in mammalian hepatocytes, this strongly suggests that the insulin signaling pathway might control the expression of the *hmgcr* gene. In hepatocytes, insulin is reported to act on *hmgcr* transcription through a helix-loop-helix transcription factor called SREBP-1c (Sterol Response Element Binding Protein) [40]. Although three SREBP isoforms have been identified, it appears that only the SREBP-1c form responds to insulin. The two others are more sensitive to cholesterol, which inhibits *hmgcr* gene expression through a negative feed back loop. In *Drosophila*, only one SREBP homolog has been identified: *hlh-106* (dSREBP) [49], [50]. In a recent study, dSREBP was found to be expressed in *ca* cells [49]. We used DI-3 and DI-11 lines to drive a dominant negative form of this gene: p[UAS-*dsrebp^DN^*] (UAS-*dsrebp^DN^*) in the *ca* and observe a lack of sexual dimorphism (Figure S1). Furthermore, when using DI-3 to drive the UAS-*dsrebp^DN^* cDNA gene, the sexual dimorphism was retrieved in flies older than 5 days. These results resemble those obtained after the disturbance of InR or HMGCR expression in the *ca*. SREBP may therefore be a molecular link between InR and HMGCR in *ca* (Figure 8).

### Body size depends of the *hmgcr*

In *Drosophila*, the insulin signaling pathway regulates growth in accordance with the nutritional availability during development [7], [16], [26], [27]. Disruption of various components of this pathway leads to delayed and small (dwarf) flies [6], [8], [12], [16]. However, very little is known about the precise tissue specificity of the insulin pathway's action requirement. In fact, in contrast to mammals which display a wide variety of growth factors [60], the predominant role of insulin pathway in development points to an action occurring in all cells of the organism [16], [17], [26]. However, in some cases, some organs seem to be more or less affected compared to others [61], while a recent study showed dwarf and delayed larvae in flies lacking SREBP in some specific tissues [49]. In certain temperature conditions, we observed delayed and small (dwarf) flies by disrupting InR and/or HMGCR expression or using dominant negative forms of SREBP specifically targeted in the *ca*. Taken together, these results strongly suggest a crucial role of these components (InR-HMGCR-SREBP), specifically in the *ca* for controlling body size. Although these components are linked to the insulin pathway, our results suggest a new mechanism, probably involving neuroendocrine control by JH, which would be independent of the IGF-like role of insulin occurring in all cells, to determine the body size.

### The sexual dimorphism in locomotor activity

We showed that HMGCR and InR (as well as the first results obtained with SREBP), specifically within the *ca*, are implicated in the control of the sexual dimorphism. Moreover, we have not detected any sexual difference neither in InR, nor in HMGCR expression pattern or in the efficiency of *inr* and/or *hmgcr* RNAi silencing, suggesting that the sexual dimorphism in locomotor activity does not rely in the expression pattern of these genes. Previous studies have shown the implication of some *PI* neurons in the control of the sexual dimorphism, as those neurons have been feminized by the directed expression, in males, of the female version of the sexual determination factor *transformer* (*tra*), leading to name those cells “feminizing cells” (FCs) [28], [29]. Moreover, we have shown that these cells act on the sexual dimorphism through a neuroendocrine factor, by regulating the synthesis or JH release, probably by acting on the *ca*'s cells. Here, in this study, we observed that the directed expression of *tra* in the IPCs does not influence the sexual dimorphism (data not shown). Thus, it is likely that two independent mechanisms could control the sexual dimorphism. The first, composed by the FCs, might act on the cells of the *ca* and consequently on the JH. This could be qualified as the “informative” way. The second way composed by the IPCs, might act on the cells of the *ca* that express the InR and HMGCR components (Figure 8). This could be qualified as the “permissive” way that allow the HMGCR activity and consequently, the synthesis of the JH. In brief, the informative way may control the level of synthesis and/or the timing of JH release, while the permissive way could allow the synthesis of JH. This could suggest that the informative, but not the permissive way is *tra*-sensitive. Additionally, this could also suggest that the disruption of the permissive way abolishes the sexual dimorphism. The precise interaction between these two putative ways still remains to be thoroughly characterized.

In parallel, other studies have demonstrated that disrupting the insulin pathway increases longevity [13], [15], [62], while in some cases, it also disrupts the sexual dimorphism [21]. It will be relevant to determine if the inactivation of the expression of either the *inr* and *hmgcr* genes, specifically in the *ca*, using the RNAi technology (DI-11/RNAi-InR and DI-11/RNAi-HMGCR) could also increase longevity. In summary, we investigated the role of the HMGCR in adult flies in these studies and find a functionally conserved link between InR and HMGCR. This could lead to the establishment of a new model to study the molecular and physiological roles of the reductase in a relatively simple organism.

## Materials and Methods

### Flies stock

All *Drosophila melanogaster* lines were maintained at 24°C on standard food medium. Wild-type Canton Special (CS) flies were used. P[GAL4]C316 was kindly provided by S. Waddell [58] and p[UAS-*clb*] by R. Lehmann [45]. *srebp^DN^*, *hmgcr^11635^* (P{PZ}l(3)04684^04684^), p[UAS-*gfp*], p[elav-GAL4], p[daughterless-GAL] and p[actin-GAL4] lines were obtained from the Bloomington *Drosophila* Stock Centre. p[UAS-RNAi-InR] (18402-R1 and 18402-R2) and p[UAS-RNAi-HMGCR] (10367-R1 and 10367-R3) were kindly provided by R. Ueda (NIG, Japan).

### Quantification of locomotor activity (start/stop number)

A previously described paradigm was used [28], [30]. Two, four, or five days old flies were allowed to walk in a small square arena (4×4 cm, 3.5 mm high) for five hours. A camera placed above the arena recorded fly movements (Ethovision, Noldus, Netherland). The number of activity and inactivity phases (also equivalent to the starts/stops number) was quantified by the computer software. Since in some cases, the level of locomotor activity differs among groups with different genotypes, the number of start/stop was normalized by designating the females of each group as the reference (expressed as 100%) and comparing males in the same group to the females. Flies and their respective controls were always recorded at the same time. All experiments were performed at 24°C and 60% humidity. Statistical comparisons were made with ANOVA tests, using Statistica software (StatSoft, Inc.).

### Genesis of the p[hmgcr-GAL4] construct and *Drosophila* transgenic flies lines

To generate the p[hmgcr-GAL4] lines, we amplified by PCR, two DNA fragments from wild-type CS, using a long range Taq DNA polymerase (Invitrogen Long Range) according with the fabricant protocol. The first fragment corresponds to the putative promoter region controlling the transcription of the RNA_A_. For this fragment (3857 pb length: −3569 bp to +288 bp from the start site of the *hmgcr* gene), we used two primers flanked by a SfiI (SFI-RB4) and EcoRI (Eco-RB5) restriction site to allow the insertion in the pChsGAL4 plasmid. (SFI-RB4: ACGGCCTATGCGGCCCAGACGGTGAGTACAACGTA; Eco-RB5: ACGAATTCGTCTAGAGCGACTGCCAATT). The second fragment corresponds to the putative promoter region controlling the transcription of the RNA_B_. In order to amplify this second fragment (10792 pb length: +2733 bp to +13525 bp from the start site of the *hmgcr* gene), we used two primers also flanked by a SfiI (SFI-RB1) and EcoRI (Eco-RB2). (SFI-RB1: ACGGCCTATGCGGCCCCAGCTCCAACATGATGCTA; Eco-RB2: ACGAATTCCCTTCGGTTTCTACGCACTT) restriction site. After digestion (by SfiI and EcoRI) and purification, each fragment was inserted into the pChsGAL4 plasmid. After transformation of JM109 bacteria, we amplified, extracted, and purified the plasmids using a Midiprep Kit (Quiagen). We then injected each construct/plasmid with a helper plasmid in embryos (w-) Canton-S and selected transgenic flies. Two independent transgenic lines carrying the putative RNA_A_ promoter were obtained and named DI-3-2 and DI-3-3. Both independent insertions, homozygous viable, are on the second chromosome and present the same expression pattern. Thus, although all experiments have been performed with both lines, since both lines give similar results, the results reported here are from the line DI-3-3 (stated DI-3 in the text). One transgenic line carrying the putative RNA_B_ promoter was obtained and named DI-11. The insertion site is on the third chromosome, and is homozygous lethal.

### Immunohistochemical techniques

Adult *Drosophila* heads were fixed (Carnoy) for mass histology (paraffin section) or in 4% paraformaldehyde (PFA) (for cryostat section) as described in Belgacem and Martin (2006) [21]. Seven-micrometer sections were blocked for one hour in normal horse serum (PBT_0,05_: PBS+0.05% Triton X-100). A primary anti-HMGCR antibody raised from the human form (1/20 in PBT_0,05_) (rabbit polyclonal antibody) (a courtesy of P. Edwards [33]), was added, and incubated overnight at 4°C. After three washes in PBT_0,05_, a secondary antibody Cy3-labeled anti-rabbit (1/500 in PBT_0,05_; Amersham) was applied for one hour, at room temperature. After three washes in PBT_0,05_ and one in PBS, slices were mounted in DABKO and observed by fluorescence microscopy.

*Cardia* and *corpus cardiacum*/*corpus allatum* were dissected in 4% PFA at 4°C, incubated 30 min and washed in PBS (4°C) and twice in PBT_0,05_. For double immunostaining (HMGCR and InR), a primary anti-HMGCR antibody (1/20 in PBT_0,05_) (rabbit polyclonal antibody) and a primary anti-InR antibody (1/20 in PBT_0,05_) (a mouse monoclonal antibody against the human InR α-subunit; Chemicon) were added, and incubated overnight at 4°C. For double immunostaining (HMGCR and GFP), the primary anti-HMGCR antibody (1/20 in PBT_0,05_) and a primary anti-GFP mouse antibody (1/1000 in PBT_0,05_; Roche) were added, and incubated overnight at 4°C. After three washes in PBS, the first antibodies were recognized by Cy3-labeled anti-rabbit secondary antibody (1/500 in PBT_0,05_; Amersham) for anti-HMGCR, and FITC-conjugated anti-mouse (1/500 in PBT_0,05_; Sigma) secondary antibodies for anti-GFP or anti-InR. After three washes in PBT_0,05_ and one in PBS, slices were mounted in DABKO and observed by fluorescence microscopy.

For the double immunostaining (AKH and GFP), *cardia* and *corpus cardiacum*/*corpus allatum*, have been prepared as described above, except for antibodies. Primary antibodies, anti-HMGCR (1/20 in PBT_0,05_) and anti-AKH labeled with rhodamine (1/800 in PBT_0,05_) [56] were added and, after washes (in PBT_0,05_), anti-HMGCR (Rabbit ) were recognized by FITC-conjugated anti-mouse (1/500 in PBT_0,05_; Sigma) secondary antibody.

## Supporting Information

Figure S1 Targeted expression of a dominant negative form of SREBP in the *corpus allatum* abolishes the sexual dimorphism. Expressing p[UAS-*srebp^DN^*] in the *ca* under the control of DI-11 abolishes the sexual dimorphism (b) comparing to appropriated controls p[UAS-*srebp^DN^*]/CS (a).
(0.06 MB TIF)
