# Supplementary figures and images for: Hmgcr in the Corpus Allatum Controls Sexual Dimorphism of Locomotor Activity and Body Size via the Insulin Pathway in Drosophila
Source: PLoS One. 2007 Jan 31;2(1):e187. doi: 10.1371/journal.pone.0000187 (PMC1779623; doi:10.1371/journal.pone.0000187)

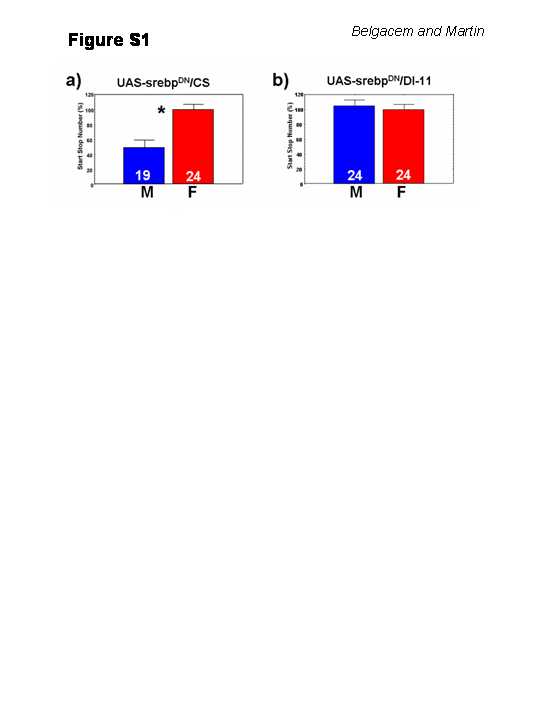

Supplement: Figure S1 — Targeted expression of a dominant negative form of SREBP in the corpus allatum abolishes the sexual dimorphism. Expressing p[UAS-srebpDN] in the ca under the control of DI-11 abolishes the sexual dimorphism (b) comparing to appropriated controls p[UAS-srebpDN]/CS (a). (0.06 MB TIF) [file pone.0000187.s001.tif]
